# Systematic review and meta-analysis of the antioxidant capacity of lycopene in the treatment of periodontal disease

**DOI:** 10.3389/fbioe.2023.1309851

**Published:** 2024-01-08

**Authors:** Nansi López-Valverde, Antonio López-Valverde, Bruno Macedo de Sousa, José Antonio Blanco Rueda

**Affiliations:** ^1^ Department of Medicine and Medical Specialties, Faculty of Health Sciences, Universidad Alcalá de Henares, Madrid, Spain; ^2^ Department of Surgery, University of Salamanca, Instituto de Investigación Biomédica de Salamanca (IBSAL), Salamanca, Spain; ^3^ Institute for Occlusion and Orofacial Pain Faculty of Medicine, University of Coimbra, Polo I‐Edifício Central Rua Larga, Coimbra, Portugal

**Keywords:** lycopene, antioxidant, periodontal disease, gingivitis, periodontitis, meta-analysis

## Abstract

This systematic review with meta-analysis evaluated the antioxidant effect of lycopene as an adjuvant treatment for periodontal disease. PubMed, EMBASE and Web of Science databases were consulted. According to the PICOs strategy, inclusion criteria were established for intervention studies Randomized Controlled Trials in Probing depth subjects (participants) treated with conventional treatment and lycopene (intervention) compared to patients treated with conventional treatment (control) in which periodontal response to treatment (outcome) was assessed. The risk of bias for randomized studies was assessed using the Cochrane Risk of Bias Tool. The methodological quality of the studies included in the meta-analysis was measured using the Jadad scale. Quantitative data were analyzed using six random-effects meta-analyses, taking into account periodontal parameters: Probing Pocket Depth, Clinical Attachment Loss, Bleeding on Probing, Plaque Index, Uric Acid and Gingival Index. Six further meta-analyses were performed, according to the follow-up of the studies (short-, medium- and long-term). Of the 339 studies identified, only 7 met the eligibility criteria. The meta-analysis of the studies according to the parameters evaluated only obtained statistical significance in the assessment of plaque index (*p* = 0.003). Regarding follow-up periods, PPD was significant (*p* = 0.03) in the short term. bleeding on probing estimates were significant in the short and medium term (*p* = 0.008 and *p* = 0.03, respectively), IP was significant in the short and medium term (*p* = 0.0003 and *p* = 0.01, respectively) and gingival index in the short and medium term (*p* = 0.002 and *p* = 0.02, respectively). Heterogeneity was high (I^2^ >50%) in all assessments, except for Clinical Attachment Loss (I^2^ = 16.7%). The results demonstrate that antioxidant treatment with lycopene could be useful as an adjunctive treatment for periodontal disease.

## 1 Introduction

Periodontal diseases (gingivitis and periodontitis) (PD) are a group of chronic multifactorial inflammatory pathologies, associated with biofilms and destructive of the supporting tissues of the tooth. (Papapanou et al., 2018).

Gingivitis is considered the first stage of the disease and is mainly manifested by bleeding gums. If left untreated, it evolves over time to periodontitis, with the consequent accumulation of dental biofilm, gum recession, pocket formation, bacterial dysbiosis and, finally, destruction of the supporting tissues of the tooth, leading, eventually, to its loss (Albandar, 2005).

*Porphyiromonas gingivalis* (*P. gingivalis*), *Treponema denticola* (*T. denticola*) and *Tannerella forsythia* (*T. forsythia*) are the responsible pathogens, although certain Gram-negative bacteria, such as *F. nucleatum* (*Fusobacterium nucleatum*), *A. actinomycetemcomitans* (*Aggregatibacter actinomycetemcomitans*) and some Gram-positive Streptococci play an important role in the pathogenesis of the disease (Iniesta et al., 2023). However, new concepts of periodontal pathogenesis propose that periodontitis would be initiated by a dysbiotic microbial community and not by periodontal pathogens; in this sense, accessory pathogens would act by favoring the colonization of the responsible pathogens, whereas pathobionts would contribute to the destructive inflammation (Hajishengallis and Lamont, 2014).

It is one of the most prevalent mutilating pathologies, being considered that about 11% of the world’s population suffers from it, with more than 750 million people affected. Increased life expectancy in today’s society, has transformed this pathology into a public health burden, with the consequent increase in costs for the different healthcare systems (Chen et al., 2021).

There is increasing evidence of the relationship between periodontitis and certain systemic diseases (Linden et al., 2013; Bui et al., 2019). It is well known that uncontrolled diabetes, is associated with severe periodontitis and that there is a bidirectional relationship between both pathologies, as well as that patients with uncontrolled diabetes, develop periodontal pathologies with a high destructive capacity (Negrato et al., 2013; Stöhr et al., 2021).

Cardiovascular pathologies and their correlation with PD have attracted the attention of different researchers, although a direct relationship has not been consensually demonstrated; a meta-analysis by Bahekar et al., 2007 on five cohort studies including a sample of more than 86,000 patients concluded that patients with PD had a 1.14-fold increased risk of developing coronary heart disease (Bahekar et al., 2007). Gao et al., 2021 in a recent meta-analysis including 11 retrospective studies with more than 200,000 participants showed that periodontitis was a risk factor for coronary heart disease and that the number of missing teeth would be directly correlated with the risk of coronary heart disease (Gao et al., 2021).

Certain pathogens from the dental biofilm of patients with PD, such as *P. gingivalis* and some types of *Streptococci*, have been implicated in pulmonary infections by an aspiration mechanism (Gomes-Filho et al., 2014); even Heo et al., 2011 demonstrated the existence of genetically similar strains of *C. Albicans* in samples from dental biofilm and tracheobronchial secretions from bronchoalveolar lavage, of the same patient (Heo et al., 2011). Something similar would occur with *Chlamidia Pneumoniae* (*C. Pneumoniae*), a pathogen associated with chronic obstructive pulmonary disease (COPD), found in the bacterial plaque of patients with periodontitis (Almeida-da-Silva et al., 2019). An interconexion between PD and neurodegenerative diseases, autoimmune diseases and certain types of cancer has also been demonstrated, although a recent review conducted recently by Hajishengallis, 2022 reported that, despite current knowledge, no unequivocal evidence is available that effective treatment of periodontitis, can improve the risk or incidence of epidemiologically related comorbidities (Hajishengal and lis, 2022).

In addition to the microbial component, host immunoinflammatory susceptibility plays a relevant role (Cekici et al., 2014). Oxidative stress (OS) generated by the imbalance between the oxidative load and the antioxidant capacity of the host is considered decisive in the progression of PD (Avezov et al., 2015). There is increasing evidence that reactive oxygen species (ROS) play an important role in PD-associated tissue damage (Nibali and Donos, 2013; Wang et al., 2017), so that the use of antioxidants in the treatment of PD would be sufficiently justified. Enzymatic and non-enzymatic antioxidants have been investigated and described. The former are intrinsic in the human body and produce a direct neutralization of ROS and they are constituted by primary enzymes involved in the protection of the human organism, in an attempt to maintain ROS levels in normal ranges (Battino et al., 1999). Non-enzymatic ones are exogenous and are represented by fat-soluble vitamins, water-soluble vitamins flavonoids and trace elements and the organism obtains them through balanced diets with an abundance of vegetables and fruits (Prior, 2003).

Certain natural antioxidants have shown efficacy in the treatment of PD (Kamodyova et al., 2013) and regular consumption of natural carotenoids has been reported to protect against OS by modulating immune and inflammatory markers; a randomized crossover study in 26 individuals on a carotenoid-supplemented diet found a significant reduction in tumor necrosis factor-alpha (TNF- α) and interleukin IL-1, two biomarkers of great importance in the monitoring of PD (Riso et al., 2006).

Lycopene is a lipophilic carotenoid, a natural antioxidant, found in certain vegetables and fruits, such as tomatoes, grapes, watermelons, papayas, and blueberries (Li N. et al., 2021). Different properties have been attributed to it, such as anticarcinogenic, cardioprotective, anti-inflammatory, antihypertensive, and above all, a potent antioxidant action (Leh and Lee, 2022). Precisely, this potent antioxidant action is associated with a lower risk of chronic diseases (Rao and Rao, 2007) and it has been shown that high concentrations of lycopene in serum, are associated with lipid peroxidation and a decrease in protein oxidation (Mackinnon et al., 2011). At the cellular level it has been shown that, lycopene, has proliferative effects on osteoblasts, increasing bone regeneration, as well as an inhibitory effect on osteoclastic formation and resorption, which could be very useful in tissue engineering, since lycopene could increase the quality and speed of new bone formation in periodontal treatments (Sołtysiak and Folwarczna, 2015; Bengi et al., 2023).

Therefore, there seems to be an interrelation between low antioxidant levels and PD and the aim of our meta-analysis was to evaluate, in randomized clinical studies, the antioxidant effect of lycopene in the treatment of this pathology.

## 2 Materials and methods

### 2.1 Study design and registration

This study is presented in accordance with the PRISMA (Preferred Reporting Items for Systematic Reviews and Meta-Analyses) statement (Page et al., 2021) and the guidelines of the Clinical Practice Guidelines (Graham et al., 2011).

The protocol of our meta-analysis was registered in INPLASY with the number INPLASY202390106 (DOI: 10.37766/inplasy2023.9.0106).

### 2.2 Question of interest

The research question was formulated according to the PICOS strategy: “In patients with PD, does the antioxidant action of lycopene have a clinically significant additional effect when used alone or as an adjuvant to conventional treatment? Interventional studies in adult humans with PD (P) comparing conventional periodontal treatment with the addition of lycopene (I) *versus* patients who had only received conventional periodontal treatment (C) were included to observe the effects of periodontal treatment (O); only randomized clinical studies (S) were considered (Table 1).

**TABLE 1:** Search strategy and the PICOS format.

Population	Subjects with periodontal disease
Intervention	Conventional periodontal treatment + lycopene
Comparisons	Conventional periodontal treatment
Outcomes	To observe the effects of treatment on biomarkers indicative of PD and/or values of antioxidant substances (ΔPPD, ΔCAL, ΔBOP, ΔPI, ΔUA and ΔGI)
Study design	Randomized Controlled Trials (RCTs)
Search combination	#1 AND #2 OR
Language	English
Electronic databases	PubMed/MEDLINE; WOS; EMBASE

PPD, probing pocket depth; CAL, clinical attachment loss; BOP, bleeding on probing; PI, plaque index; UA, uric acid; GI, gingival index; Δ, Values achieved after treatment.

### 2.3 Data sources and search strategy

The electronic databases PubMed (via MEDLINE), EMBASE and Web of Science (WOS) were searched for articles published up to August 2023. The MeSH (Medical Subject Headings) terms used in the MEDLINE (PuBMed) databases were: “Anti-Inflammatory Agents” [MeSH terms], “Antioxidants” [MeSH terms]; “Carotenoids” [MeSH terms]; “Lycopene” [MeSH terms]; “Periodontal Diseases” [MeSH terms]; “Dental Plaque” [MeSH terms]; “Gingivitis” [MeSH terms]; “Periodontitis” [MeSH terms]. The search terms used in EMBASE were: “Antioxidants”; “Lycopene”; “Periodontal Diseases”; “Gingivitis”; “Periodontitis”. In WOS, the search terms were: “Antioxidants”; “Lycopene”; “Gingivitis”; “Periodontitis”. Boolean AND-OR operators were used to refine the search.

We considered that the three databases were sufficient to obtain a complete search, since the PubMed database contains more than 36 million citations and abstracts of bi-omedical literature. MEDLINE is the National Library of Medicine’s main bibliographic database and contains more than 29 million references to scientific articles, especially in biomedicine. Embase (Elsevier ed) is a database of biomedical literature, with millions of journal records and scientific communications, where it is possible to identify the role of a particular drug or product. WOS (Clarivate Analytics) is a collection of databases of bibliographic references and citations of periodicals, collecting information from 1900 to the present.

### 2.4 Inclusion and exclusion criteria

Studies were selected according to the following criteria:

Inclusion criteria.

a) RCTs (single or double-blind) conducted in patients with PD defined as bleeding, bone loss ≥2 mm and/or suppuration to peri-implant probing (≥4 mm).
b) Studies comparing the efficacy of adjuvant treatment with local/systemic lycopene versus single surgical or non-surgical treatment, in PD
c) Articles in English language.

Exclusion criteria.

a) Less than five patients per treatment group.
b) Studies assessing the efficacy of lycopene on PD associated with other systemic pa-thologies.
c) Lack of relevant or demonstrative clinical data on PD.
d) *In vitro* studies
e) Case series or clinical cases.
f) Non-relevant studies and literature reviews.

### 2.5 Study selection, data extraction and analysis

Two reviewers (NL-V and AL-V) independently compiled the titles and abstracts of the previously selected articles and entered them into an Excel spreadsheet, eliminating studies that did not refer to the research question posed. To determine the concordance between reviewers, Cohen’s kappa index (*κ*) (Cohen, 1968) was calculated and discrepancies be-tween the two, regarding the eligibility of the studies, were reviewed and discussed by a third reviewer (BM de S). Finally, the selected articles were obtained for reading, review, data extraction and inclusion. The bibliographic references of the included studies were also reviewed as an additional source of potential studies.

### 2.6 Assessment of the quality of the reports of the included studies

The methodological quality of the included studies was assessed using the Jadad Scale (Oxford quality scoring system) (Jadad et al., 1996). This validated scale, is based, fundamentally, on the description of randomization, blinding and dropouts. The scale ranges from 0 to 6; a score ≤3 means low quality of information and scores ≥4 are considered acceptable studies. Scores 5 and 6 are awarded to rigorous studies.

### 2.7 Risk of bias

NL-V and AL-V independently assessed the quality of the studies included in the meta-analysis according to the Cochrane Risk of Bias Tool (RoB2) (Minozzi et al., 2022). This tool assesses five domains of bias (randomization process, deviations from intended interventions, missing outcome data, outcome measurement, and selection of reported outcomes).

The Cochrane Hand-book for Systematic Reviews of Interventions was used. The rating “high” was given to studies considered to have a high risk of bias, “low” to those considered to have a low risk of bias, and “borderline” indicated the presence of bias due to uncertainty or lack of information on possible bias. Thus, studies were classified as having low, high or borderline risk of bias. Any discrepancies in the assessment of RoB2 were discussed between the two reviewers with the aim of reaching a consensus between them.

### 2.8 Statistical analysis of data, meta-analysis

Data from the included studies were analyzed using Review Manager software (RevMan Software. Version 5.4.1; The Cochrane Collaboration, Copenhagen, Denmark; 2020), to assess the efficacy of periodontal treatment associated with lycopene on periodontal parameters. Two meta-analyses were performed: the first according to the parameters or biomarkers investigated in the selected studies; the second according to the follow-up periods: short-term, 2- and 3-week follow-up (Belludi et al., 2013; Kaur et al., 2017); mid-term 6 and 8 weeks (Arora et al., 2013; Tripathi et al., 2019; Wasti et al., 2021) and long-term 12 and 24 weeks (Chandra et al., 2012; Tawfik et al., 2019). Both were based on mean difference (MD) and standard deviation (SD) to estimate effect size, with 95% confidence intervals (CI) to assess adverse outcomes. The random-effects model was selected taking into account the un-certainty in I2, considering the scarcity of studies and the methodological heterogeneity found in the included studies. Heterogeneity was considered low with I^2^ = 25%, moderate, I^2^ = 50% and high I^2^ = 75%. The threshold for statistical significance was established as *p* < 0.05. A meta-analysis of adverse outcomes was not performed due to lack of data reporting.

## 3 Results

### 3.1 Characteristics of the studies. qualitative summary

**FIGURE 1:**
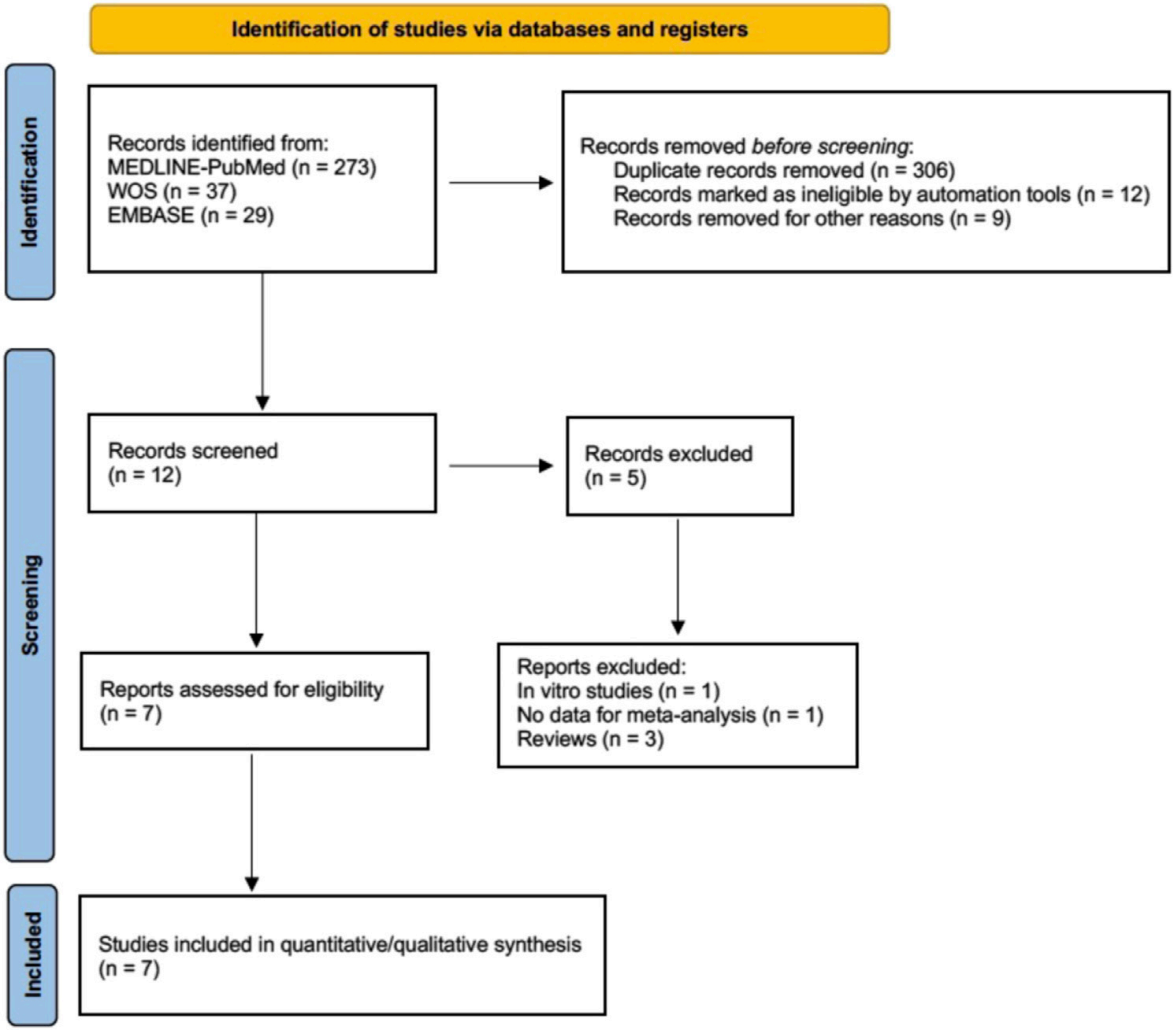
Flow diagram.

After exclusion, 7 studies were finally selected and collected for meta-analysis (Chandra et al., 2012; Arora et al., 2013; Belludi et al., 2013; Kaur et al., 2017; Tawfik et al., 2019; Tripathi et al., 2019; Wasti et al., 2021) (Figure 1, Flow diagram).

The discrepancy between the NL-V and AL-V reviewers was only 15%, which resulted in a high level of reviewer concordance (*κ* = 85%).

The seven studies selected for meta-analysis included 316 subjects. The studies by Chandra et al., 2012 (Chandra et al., 2012) and Kaur et al., 2017 (Kaur et al., 2017) had the largest sample sizes, with 100 and 60 subjects, respectively; the study by Tawfik et al., 2019 (Tawfik et al., 2019) had the smallest sample size, with only 16 subjects. Follow-up of the studies ranged from 3 (Belludi et al., 2013; Kaur et al., 2017) to 24 weeks (Tawfik et al., 2019). Probing depth (PD) was reported by 5 studies (Chandra et al., 2012; Arora et al., 2013; Belludi et al., 2013; Kaur et al., 2017; Tawfik et al., 2019); clinical attachment loss (CAL) was re-ported by 4 studies (Chandra et al., 2012; Arora et al., 2013; Belludi et al., 2013; Tawfik et al., 2019); bleeding on probing (BOP), 4 studies (Arora et al., 2013; Belludi et al., 2013; Tripathi et al., 2019; Wasti et al., 2021); plaque index (PI), 6 studies (Chandra et al., 2012; Arora et al., 2013; Kaur et al., 2017; Tawfik et al., 2019; Tripathi et al., 2019; Wasti et al., 2021) and gingival index [GI], 4 studies (Chandra et al., 2012; Arora et al., 2013; Kaur et al., 2017; Tawfik et al., 2019). Thus, PI was the most reported periodontal index in the studies included in the meta-analysis.

All of the studies were conducted at Indian research centers, except that of Tawfik et al., 2019 (Tawfik et al., 2019).

Which was conducted by Egyptian researchers. The age range of the subjects included in the studies ranged from 18 to 55 years. Five studies resorted to systemic treatment and only two to local use of the antioxidant. Student’s t-test or *t*-test was the most commonly used statistical determination. The general and specific characteristics of the included studies are shown in Table 2 and Table 3.

**TABLE 2:** General characteristics of the studies included in the meta-analysis.

Study	Aim	Participants; groups	Interventions	Follow-up	Clinical parameters assessed	Conclusions
Belludi et al. (Belludi et al., 2013)	To evaluate the effect of lycopene as an adjunct to mechanical therapy in the treatment of periodontal disease gingivitis and periodontitis	20 participants 2 groups	Group 1: SRP Group 2: SRP with lycopene	21 days	PPD, CAL, BOP	Lycopene is a promising treatment as an adjunct to full oral cavity SRP in patients with moderate periodontal disease
Chandra et al. (Chandra et al., 2012)	To evaluate the efficacy of locally administered antioxidant lycopene-gel on periodontal health	100 participants 2 groups	Group 1: SRP with placebo Group 2: SRP with lycopene	12 and 24 weeks	PPD, CAL, PI, GI	Lycopene gel formulation is effective in increasing clinical fixation
Arora et al. (Arora et al., 2013)	To determine whether daily dietary supplementation for 2 months with lycopene in addition to non-surgical mechanical periodontal therapy improves clinical and immunological parameters in chronic periodontitis	42 participants 2 groups	Test Group: Lycopene Placebo Group Adjunctive SRP	8 weeks	PPD, CAL, BOP, PI, GI, UA	The lycopene group showed better results compared to the placebo group with reference to PI, GI, BOP and UA levels. CAL gain and PPD reduction were not statistically significant, but showed an improvement compared to the placebo group
Tawfik et al. (Tawfik et al., 2019)	To evaluate the antioxidant effect of lycopene on changes in clinical parameters of chronic periodontitis	16participants 2 groups	Group I was treated with scaling and root planing (SRP) and local administration of lycopene, group II was treated with SRP only	24 weeks	PPD, CAL, PI, UI, GI	Lycopene administered locally together with SRP has a protective effect on the periodontal apparatus and decreases oxidative damage to proteins in the diseased periodontium
Wasti et al. (Wasti et al., 2021)	To investigate the effect of antioxidant therapy with lycopene on the progression of periodontal disease	48 participants 2 groups	In both groups, a full-mouth PRS was performed and oral hygiene instructions were given The test group received systemic (oral) lycopene	6 weeks	BOP, PPD, UI	Oral supplementation with lycopene is positively associated with salivary uric acid levels and plays an important role in the treatment of periodontal disease
Tripathi et al. (Tripathi et al., 2019)	To investigate the antioxidant influence of lycopene on periodontal health and salivary uric acid levels in patients with gingivitis as an adjunct to scaling and root planing	30 participants 2 groups	Control group received oral whole mouth prophylaxis, while participants in the test group received oral lycopene	6 weeks	BOP, PPD, UI	Lycopene may prove to be a promising prophylactic and adjunctive therapeutic modality in the treatment of patients with gingivitis
Kaur et al. (Kaur et al., 2017)	To evaluate the effects of lycopene, administered systemically, as an adjunct to scaling and root planing in patients with moderate gingivitis	60 participants 2 groups	Lycopene antioxidant therapy test group together with SRP during and SRP alone control group	3 weeks	PPD, GI, PI	systemically administered lycopene may cause better resolution of inflammation when used as an adjunct to SRP

SRP, scaling and root planing; PPD, probing pocket depth; CAL, clinical attachment loss; BOP, bleeding on probing; PI, plaque index; GI, gingival index; UA, uric acid.

**TABLE 3:** Specific characteristics of the studies included.

Study	Country	Gender	Age range	Treatment modality	Lycopene product administered	Statistical method
Belludi et al. (Belludi et al., 2013)	India	NR	30 ± 41.6 years	Systemic therapy	Lycopene (Lycotas, Pharma. Co.)	*t*-test
Chandra et al. (Chandra et al., 2012)	India	NR	25–50 years	Local delivery	Lycopene (IBYS CHEMIE International)	Bonferroni correction, *t*-test.
Arora et al. (Arora et al., 2013)	India	Men and women	25–52 years	Systemic therapy	LycoRed, Jagsonpal Pharmaceuticals	*t*-test, Student’s independent *t*- test
Tawfik et al. (Tawfik et al., 2019)	Egypt	Men and women	33–52 years	Local delivery	Lycopene Nanjing Zelang Medical Technology Co.	Kolmogorov–Smirnov and Shapiro–Wilk tests
Wasti et al. (Wasti et al., 2021)	India	NR	NR	Systemic therapy	CLIK^®^ (Idem Healthcare Pvt. Limited)	Pearson’s Chi-square test, *t*-test.
Tripathi et al. (Tripathi et al., 2019)	India	NR	18–40 years	Systemic therapy	CLIK^®^ (Idem Healthcare Pvt. Limited)	*t*-test
Kaur et al. (Kaur et al., 2017)	India	NR	18–55 years	Systemic therapy	NR	NR

NR, does Not Report.

### 3.2 Methodological assessment of studies

According to the Jadad scale, the studies by Belludi et al., 2013 (Belludi et al., 2013), Chandra et al., 2012 (Chandra et al., 2012) and Arora et al., 2013 (Arora et al., 2013) were considered rigorous studies with a high methodological quality (≥5); the studies by Tawfik et al., 2019 (Tawfik et al., 2019), Wasti et al., 2021 (Wasti et al., 2021), Tripathi et al., 2019 (Tripathi et al., 2019) and Kaur et al., 2017 [341], with scores ≤2 were considered of low methodological quality (Table 4).

**TABLE 4:** Quality score of the randomized controlled trials included in the meta-analysis, according to the Jadad scale.

Study	Randomization	Adequate Randomization method	Blinding	Double blinding	Appropriate blinding method	Dropouts	Total, score
Belludi et al. (Belludi et al., 2013)	1	1	1	1	1	DNR	5(^a^)
Chandra et al. (Chandra et al., 2012)	1	1	1	0	1	1	5(^a^)
Arora et al. (Arora et al., 2013)	1	1	1	1	1	1	6(a)
Tawfik et al. (Tawfik et al., 2019)	1	1	0	0	0	DNR	2
Wasti et al. (Wasti et al., 2021)	1	1	0	0	0	DNR	2
Tripathi et al. (Tripathi et al., 2019)	1	1	0	0	0	DNR	2
Kaur et al. (Kaur et al., 2017)	1	1	0	0	0	DNR	2

Each study was assigned a score of 0–6. Mode value: 24 ± 1.812. DNR, does not report.

^a^ Rigorous study.

### 3.3 Risk of bias assessment

**FIGURE 2:**
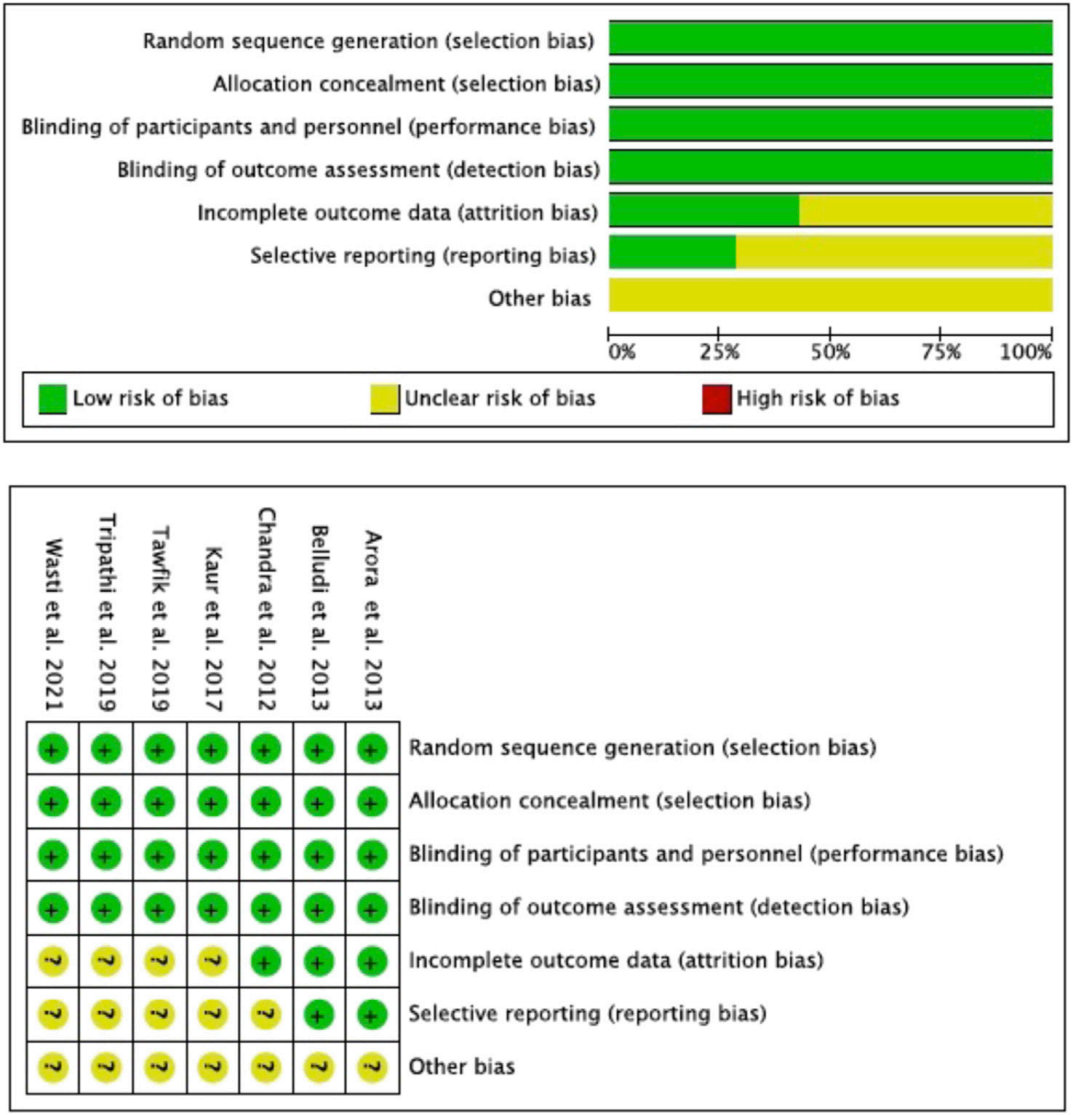
Risk of bias (Rob2) according Cochrane Hand-book.

According to the Cochrane Risk of Bias Tool (RoB2), all the studies included in the meta-analysis (Chandra et al., 2012; Arora et al., 2013; Belludi et al., 2013; Kaur et al., 2017; Tawfik et al., 2019; Tripathi et al., 2019; Wasti et al., 2021) met the domains “random sequence generation” (selection bias), “blinding of participants and personnel” (performance bias), “allocation concealment” (selection bias) and “blinding of outcome assessment” (detection bias). The domains “incomplete outcome data” (attrition bias) and “selective reporting” (reporting bias) were met by only two studies (Arora et al., 2013; Belludi et al., 2013). None of the studies reported the domain “other bias” (Figure 2).

### 3.4 Meta-analysis

#### 3.4.1 Meta-analysis of the included studies according to the parameter evaluated

**FIGURE 3:**
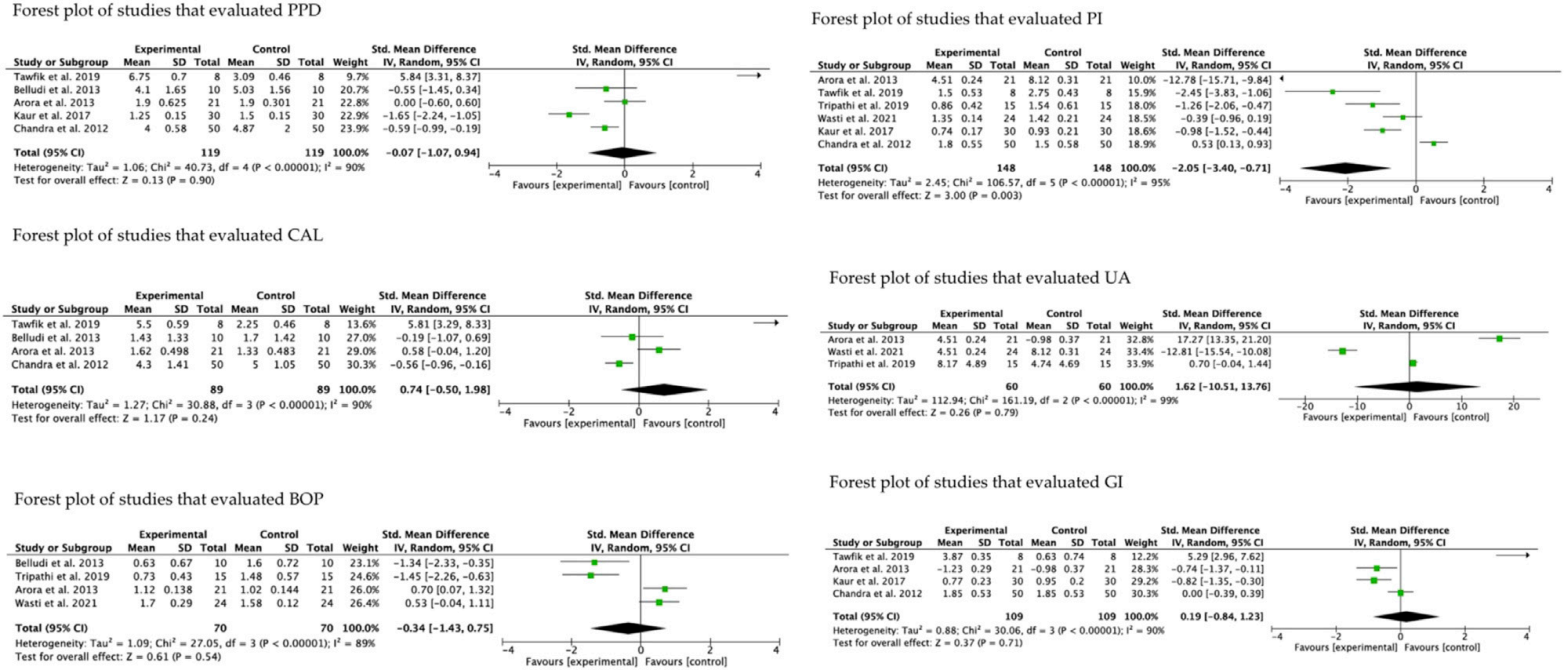
Forest plot of according to the evaluated parameters.

PPD was evaluated by five studies (Chandra et al., 2012; Arora et al., 2013; Belludi et al., 2013; Kaur et al., 2017; Tawfik et al., 2019), three of which were in favor of the experimental group (Chandra et al., 2012; Belludi et al., 2013; Kaur et al., 2017), without statistical significance (*p* = 0.90). CAL was evaluated by four studies (Chandra et al., 2012; Arora et al., 2013; Belludi et al., 2013; Kaur et al., 2017), although only two (Arora et al., 2013; Belludi et al., 2013) were in favor of the experimental group without statistical significance (*p* = 0.24). Similarly, the group that evaluated BOP (Arora et al., 2013; Belludi et al., 2013; Tripathi et al., 2019; Wasti et al., 2021), obtained 2 studies in favor of the intervention (Belludi et al., 2013; Tripathi et al., 2019) without statistical significance (*p* = 0.13). PI was evaluated by six studies (Chandra et al., 2012; Arora et al., 2013; Kaur et al., 2017; Tawfik et al., 2019; Tripathi et al., 2019; Wasti et al., 2021) and was the only group where the intervention obtained statistical significance (*p* = 0.003), with 5 studies in favor of the intervention (Arora et al., 2013; Kaur et al., 2017; Tawfik et al., 2019; Tripathi et al., 2019; Wasti et al., 2021). In the group of studies that evaluated UA (Arora et al., 2013; Tripathi et al., 2019; Wasti et al., 2021), only the study by Wasti et al., 2021 (Wasti et al., 2021) was in favor of the intervention but without statistical significance (*p* = 0.79). Finally, the group that evaluated GI (Chandra et al., 2012; Arora et al., 2013; Kaur et al., 2017; Tawfik et al., 2019), with 2 studies in favor of the experimental group (Arora et al., 2013; Kaur et al., 2017) and better performance of this group, although without es-tablished statistical significance (*p* = 0.71). Heterogeneity was high in all studies, exceeding 80% (Figure 3).

#### 3.4.2 Meta-analysis of the included studies according to follow-up times

**FIGURE 4:**
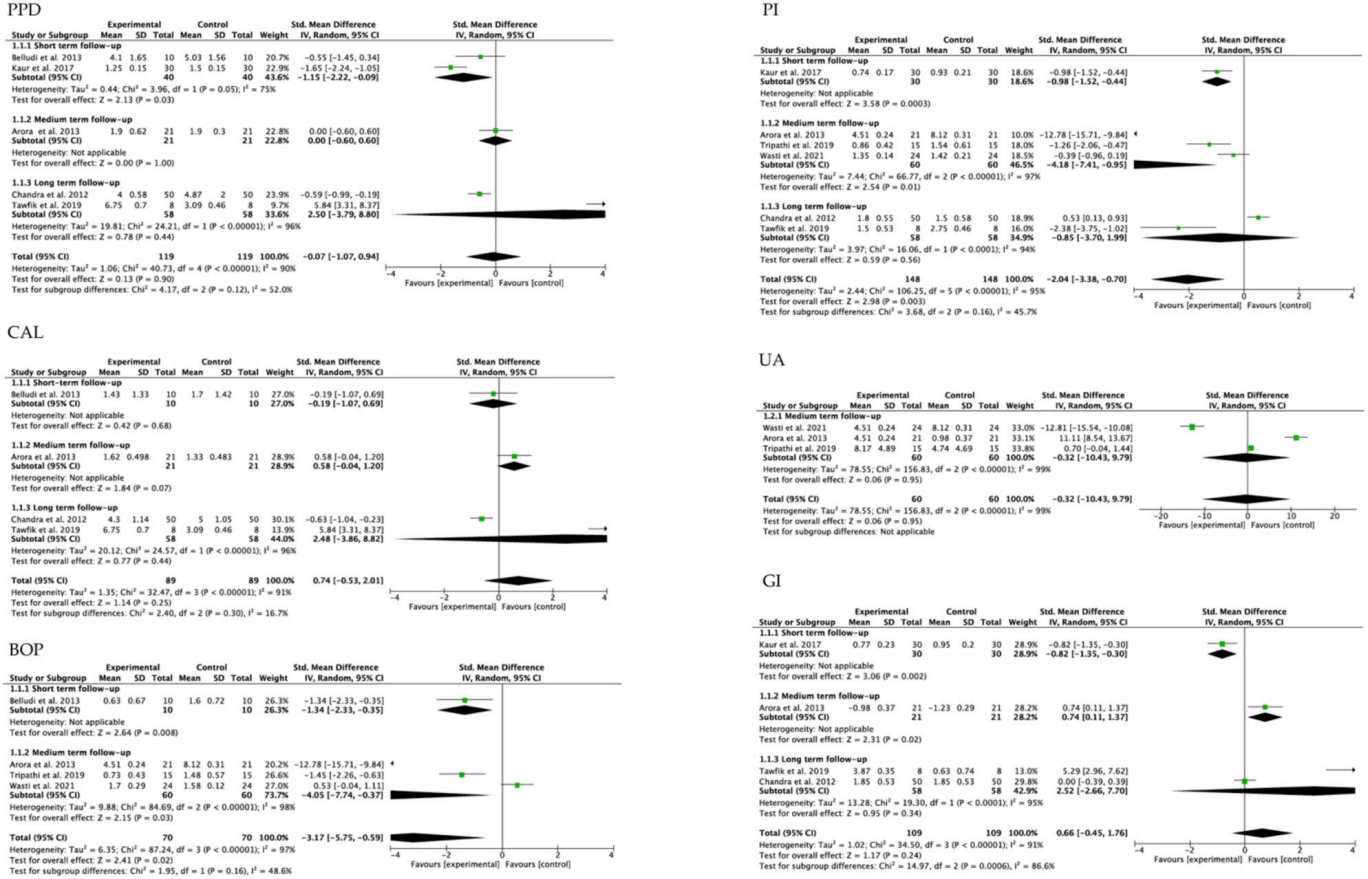
Forest plot according to follow-up period.

The analysis of the parameters evaluated in the selected studies, with respect to the follow-up periods, the PPD analysis was statistically significant (*p* = 0.03) in the short term. Similarly, BOP estimates were statistically significant in the short- and medium-term studies (*p* = 0.008 and *p* = 0.03, respectively) and PI was statistically significant in the short- and medium-term (*p* = 0.0003 and *p* = 0.01, respectively). GI assessment was statistically significant at both short-term (*p* = 0.002) and medium-term (*p* = 0.02) follow-up. Heterogeneity was low in the overall CAL assessment (I^2^ = 16.7%). All other assessments, whether short-, medium- or long-term, showed high heterogeneity >50 (Figure 4).

### 3.5 Publication bias. funnel plot

**FIGURE 5:**
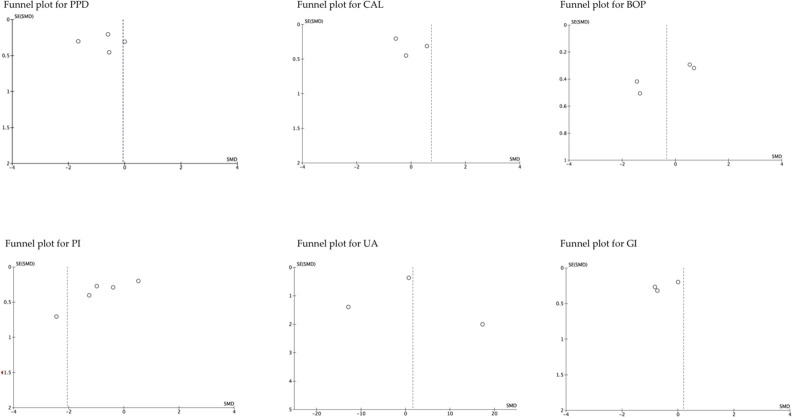
The funnel plot according to the evaluated parameters. SE, standard error; SMD, standardized mean Difference.

**FIGURE 6:**
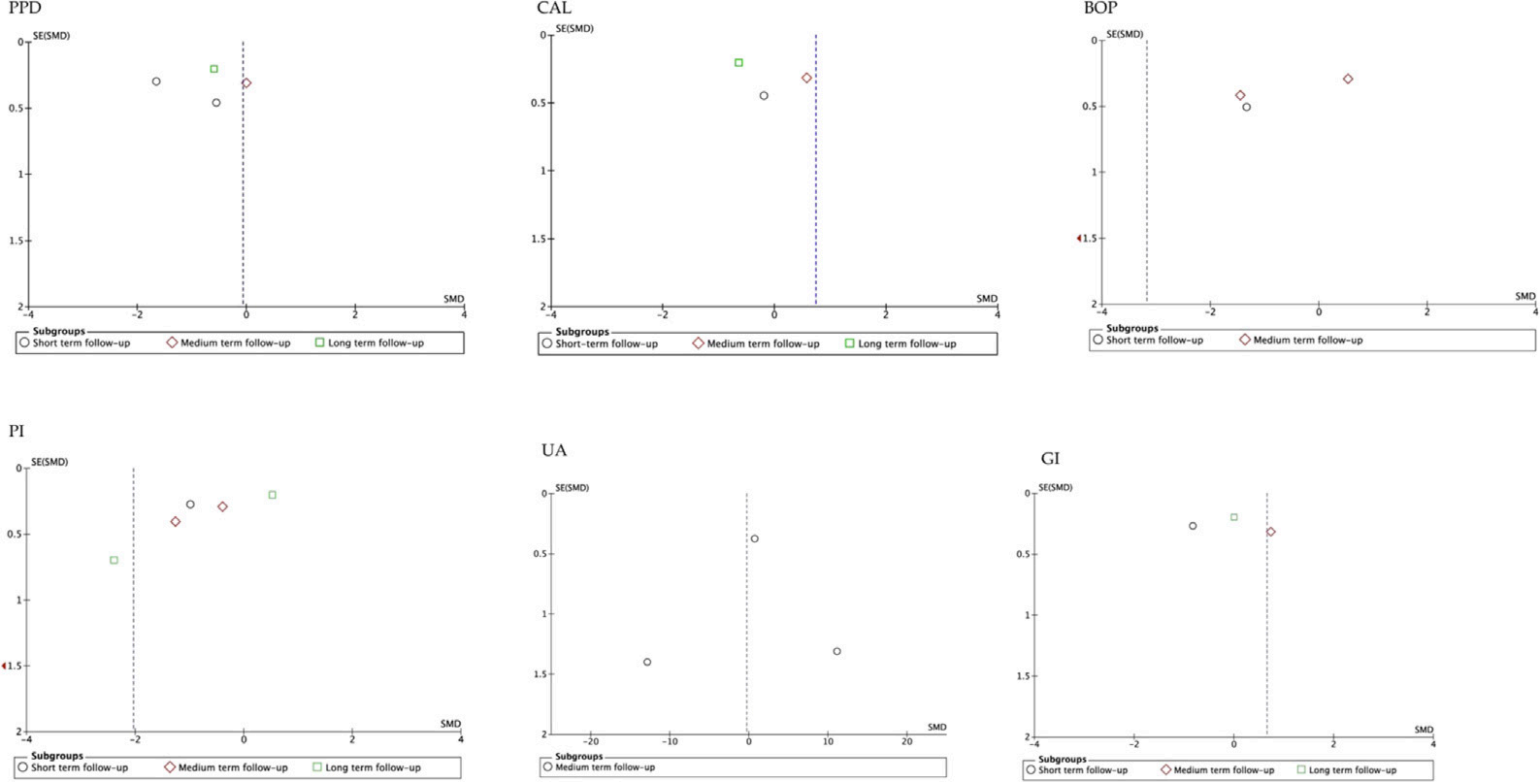
Funnel plot according to follow-up period. SE, standard error; SMD, standardized mean difference.

The funnel plot analysis of the studies that evaluated the different parameters, suggested publication biases, except those that assessed BOP (Arora et al., 2013; Belludi et al., 2013; Tripathi et al., 2019; Wasti et al., 2021). Similarly, the funnel plot according to follow-up period showed high publication bias, except for studies that evaluated medium-term UA (Arora et al., 2013; Tripathi et al., 2019; Wasti et al., 2021). In general, the estimated effect is associated with the horizontal axis and the sample size with the vertical axis (Figure 5; Figure 6).

## 4 Discussion

### 4.1 General discussion of results

In recent years, natural products have aroused great interest among the population, both in the prophylaxis and treatment of different pathologies, including oral and dental pathologies (Thomford et al., 2018; Li Y. Q. et al., 2021). Bioregulatory treatments by means of active components of medicinal plants (phytopharmaceuticals) have experienced an exponential increase in the last decades, estimating in more than 100 million dollars the worldwide economic impact of the use of phytotherapeutics in the treatment of different pathologies (World Health Organization, 2019).

Antioxidants considerably reduce the production of ROS, which is related to multiple pathologies (Ginter et al., 2014). It is known that PD can be prevented by routine hygiene strategies, although, both at the individual and public health level, they are sometimes difficult to carry out; for this reason, new products such as probiotics, vaccines and antirust agents are currently being investigated with the aim of improving prevention (Scannapieco and Gershovich, 2020). It has also been reported that ROS production is associated with increased expression of proinflammatory cytokines, responsible for bone resorption and connective tissue destruction (Imran et al., 2020).

Based on these premises, our meta-analysis evaluated the scientific evidence for the role of lycopene, a potent antioxidant in the treatment of PD, by analyzing five periodontal parameters (PPD, CAL, BOP, PI, GI) and one biomarker (UA). To achieve the objective, seven RCTs included in our study were analyzed. RCTs are considered the most rigorous studies, determining a cause-effect between a given treatment, and its results, in our case the antioxidant effect of lycopene on PD.

Apart from the antioxidant action, some studies have attributed to lycopene different cellular effects on osteoclasts, reducing their differentiation, together with a decrease in calcium phosphate reabsorption (Costa-Rodrigues et al., 2018). Systemic lycopene administration has been shown to be associated with biomarkers such as serum osteocalcin and type 1 collagen (Ardawi et al., 2016). Yoshihara et al., 2016 (Yoshihara et al., 2016) found that serum osteocalcin had a significant positive association with periodontitis and studies such as that of Golijanin et al., 2015 (Golijanin et al., 2015) showed that collagen density and volume decreased significantly as PD progressed. A recent study by Bengi et al., 2022 (Bengi et al., 2023) assessed *in vitro* the proliferative effect of lycopene on human osteoblasts, concluding that its antioxidant effect, would influence as a proliferative stimulator of osteoblastic cells, resulting in a potent bone healing agent. Another recent review reported that lycopene is a potent antioxidant and anticarcinogen, due to the modification of certain pathways that trigger cell growth or death (Ozkan et al., 2023).

Seven studies were included in our meta-analysis and all reported beneficial antioxidant effect of lycopene on PD. We found that five of the included studies resorted to systemic administration of lycopene (Arora et al., 2013; Belludi et al., 2013; Kaur et al., 2017; Tripathi et al., 2019; Wasti et al., 2021), with discordant results on its effect on the clinical parameters investigated. Belludi et al., 2013 (Belludi et al., 2013) reported a significant improvement in CAL levels (*p* = 0.043) by administration of 4 mg lycopene per day for 2 weeks. On the contrary, Arora et al., 2013 (Arora et al., 2013), with a double daily dose (8 mg), found no statistical significance for CAL values, between the lycopene-treated group and the placebo group. The beneficial effect on probing depth (PPD) was reported favorably by Belludi et al., 2013 (Belludi et al., 2013) (*p* = 0.000), and Kaur et al., 2017 (Kaur et al., 2017); Arora et al., 2013 (Arora et al., 2013) did not find statistical significance in this clinical parameter. The efficacy of the antioxidant effect of lycopene on bleeding on probing (BOP) was investigated in four of the included studies (Arora et al., 2013; Belludi et al., 2013; Tripathi et al., 2019; Wasti et al., 2021) and all agreed on favorable results on this clinical parameter.

In this regard, it has been suggested that clinical signs have relatively inconsistent sensitivity and specificity in predicting PD outcomes in untreated and treated subjects (Offenbacher, 2005) and specific biomarkers in oral fluids have been proposed as parameters of great importance for the diagnosis of PD, especially those representing inflammation, tissue degradation, and periodontal pathogens (Zhang et al., 2021). Bleeding on probing is still considered the best indicator of PD progression, while subjective methods, such as loss of adhesion and probing depth, only show past tissue destruction and do not reflect the current state of the disease (Buduneli and Kinane, 2011). Something similar occurs with the PI and GI parameters, both present great subjectivity at the time of evaluation. PI was the most evaluated clinical parameter (Chandra et al., 2012; Arora et al., 2013; Kaur et al., 2017; Tawfik et al., 2019; Tripathi et al., 2019; Wasti et al., 2021) with discordant results in the different evaluations, such as those reported by Arora et al., 2013 (*p* = 0.004) (Chandra et al., 2012), Wasti et al., 2021 (*p* = 0.002) (Arora et al., 2013), Tripathi et al., 2019 (*p* = 0.000) (Tripathi et al., 2019) and Kaur et al., 2017 (*p* = 0.000) (Kaur et al., 2017) to others such as Chandra et al., 2012 (Chandra et al., 2012) who found no differences between the lycopene treated group and the control. GI was evaluated by four studies (Chandra et al., 2012; Arora et al., 2013; Kaur et al., 2017; Tawfik et al., 2019) with equally discordant results, ranging from studies that reported statistically significant values such as Chandra et al., 2012 (Chandra et al., 2012), Tawfik et al., 2019 (Tawfik et al., 2019) and Kaur et al., 2017 (Kaur et al., 2017), to those that found no differences between the lycopene and placebo groups (Arora et al., 2013).

Which is why non-invasive, simple and reliable methods have gained ground in recent years (Podzimek et al., 2016; Melguizo-Rodríguez et al., 2020).

UA is the final product resulting from the degradation of adenine and guanine and can have both antioxidant and oxidative properties, depending on its intra- or extracellular origin (Isaka et al., 2016). Three studies (Arora et al., 2013; Tripathi et al., 2019; Wasti et al., 2021) of those included in our meta-analysis evaluated UA levels to determine whether lycopene administration increases the levels of antioxidant present in saliva, which could contribute to slowing down the destruction of periodontal tissue by free radicals. All three studies showed statistical significance (*p* = 0.02 Arora et al., 2013 (Arora et al., 2013) and *p* = 0.001 Wasti et al., 2021 and Tripathi et al., 2019 (Tripathi et al., 2019; Wasti et al., 2021)). Other studies have also shown that decreased UA levels in saliva are associated with increased severity of periodontal disease and that periodontal disease is accelerated in situations of decreased saliva antioxidant capacity and increased protein oxidation (Sculley et al., 2003; Gümüş et al., 2009).

Assessment of inflammatory mediators is of crucial importance in evaluating PD progression (Barros et al., 2016) and IL-1β and TNF-α are considered reliable biomarkers (Mogi et al., 1999). Only one of the studies included in our review (Arora et al., 2013) evaluated, in addition to clinical parameters, three inflammatory markers (IL-1β, TNF-α and UA), reporting a significant reduction of IL-1β (*p* = 0.05) in the test group compared to controls, however, they did not find a significant reduction of TNF-α levels after lycopene supplementation. The values of the biomarkers IL-1β and TNF-α, as only one study evaluated them, were not included in the meta-analysis.

### 4.2 Limitations of meta-analysis

Our meta-analysis had a number of limitations that we wish to highlight: First, the small number of RCTs included in our systematic review, and it is a statistical axiom, that sample size increases statistical power and is more representative. Secondly, the follow-up time of the included studies; we considered three types of follow-up in our meta-analysis, reduced (2–3 weeks), medium (6–8 weeks) and long-term (12–24 weeks) and this discrepancy in follow-up is a bias in obtaining results. Third, two studies (Chandra et al., 2012; Tawfik et al., 2019) administered lycopene locally and the others, systemically, with different amounts and products. Finally, it should be noted that the different statistical analyses of the data used in the included studies varied substantially.

Therefore, our results should be taken with caution.

## 5 Conclusion

The studies included in this systematic review and meta-analysis found that the antioxidant action of lycopene, either in local or systemic application, as an adjuvant to PD treatment, has a modulatory action on certain clinical periodontal parameters and inflammatory biomarkers. However, we believe that cross-sectional and multicenter RCTs (CONSORT) with large samples of subjects are warranted and necessary to confirm these results.

## Data Availability

The original contributions presented in the study are included in the article/Supplementary material, further inquiries can be directed to the corresponding author.
